# The *-KTS* splice variant of WT1 is essential for ovarian determination in mice

**DOI:** 10.1126/science.add8831

**Published:** 2023-11-02

**Authors:** Elodie P Gregoire, Marie-Cécile De Cian, Roberta Migale, Aitana Perea-Gomez, Sébastien Schaub, Natividad Bellido-Carreras, Isabelle Stévant, Chloé Mayère, Yasmine Neirijnck, Agnès Loubat, Paul Rivaud, Miriam Llorian Sopena, Simon Lachambre, Margot M. Linssen, Peter Hohenstein, Robin Lovell-Badge, Serge Nef, Frédéric Chalmel, Andreas Schedl, Marie-Christine Chaboissier

**Affiliations:** 1 Université Côte d’Azur, Inserm, CNRS, Institut de Biologie Valrose (iBV), 06108 Nice, France; 2 The Francis Crick Institute, 1 Midland Road, London NW1 1AT, UK; 3 Sorbonne Université, CNRS, Development Biology Laboratory (LBDV), 06234 Villefranche sur Mer, France; 4 Department of Genetic Medicine and Development, University of Geneva, 1211 Geneva, Switzerland; 5 iGE3, Institute of Genetics and Genomics of Geneva, University of Geneva, 1211 Geneva Switzerland; 6 Univ Rennes, Inserm, EHESP, IRSET (Institut de Recherche en Santé, Environnement et Travail)-UMR_S 1085, 35000 Rennes, France; 7 Infinity, Inserm, CNRS, University Toulouse III, 31024 Toulouse, France; 8 Central Animal and Transgenic Facility and Dept. Human Genetics, Leiden University Medical Center, 2333ZA Leiden, the Netherlands

## Abstract

Sex determination in mammals depends on the differentiation of the supporting lineage of the gonads into Sertoli or pre-granulosa cells that govern testis and ovary development, respectively. While the Y-linked testis-determining gene *Sry* has been identified, the ovarian-determining factor remains unknown. Here we identify -KTS, a major alternatively spliced isoform of the Wilms’ tumor suppressor WT1, as a key determinant of female sex determination. Loss of -KTS variants blocks gonadal differentiation in mice, whilst increased expression, as found in Frasier syndrome, induces precocious differentiation of ovaries independently of their genetic sex. In XY embryos, this antagonizes *Sry* expression, resulting in male-to-female sex reversal. Our results identify - KTS as an ovarian-determining factor and demonstrate that its time of activation is critical in gonadal sex differentiation.

In mice, sex is genetically determined by the constitution of the sex chromosomes. This leads to testis or ovary development in XY and XX embryos respectively, which in turn influences the sexual development of the whole individual. Prior to sex determination, WNT/β-catenin signaling mediated by R-spondin1(RSPO1) contributes to the proliferation of the gonadal progenitors in both sexes (1). In XY gonads, at around E(Embryonic day)11.5, RSPO1/WNT/β-catenin is downregulated and *Sry* and its direct target *Sox9* are up-regulated in a subset of progenitors derived from the overlying coelomic epithelium (2–4). These transcription factors induce Sertoli cell differentiation. Once differentiated, they no longer express *Sry* but express other genes including *Amh* (5–7) and establish the testis fate. In XX gonads, pre-granulosa cell differentiation occurs slightly later, around E12.0-12.5, as shown by their loss of bipotentiality (8–10), the de novo expression of the transcription factor FOXL2 (11), and the stabilization of RSPO1/WNT/β-catenin signaling (12, 13). However, the gene(s) initiating ovarian differentiation remained unknown (14).

One of the key factors in the early development of the gonad is the Wilms’ tumor suppressor WT1, a zinc finger transcriptional regulator (15). *WT1*(human)*/Wt1*(mouse) encodes two major alternative spliced isoforms that do or do not include the 3 amino acids KTS between the two last zinc fingers. These isoforms are named +KTS and -KTS respectively. Whilst -KTS acts as a transcriptional activator or repressor depending on the cellular context, the insertion of +KTS abrogates DNA binding and promotes the subnuclear localization of WT1 in nuclear speckles (16, 17). A simple imbalance of the ratio of both isoforms in favor of *-KTS* is the molecular basis of Frasier syndrome, characterized by male-to-female sex reversal (18, 19) associated with the downregulation of *Sry* as evidenced in the mouse model (20).

## Results

### Distribution of *-KTS* transcripts during gonadal development

**Fig. 1:**
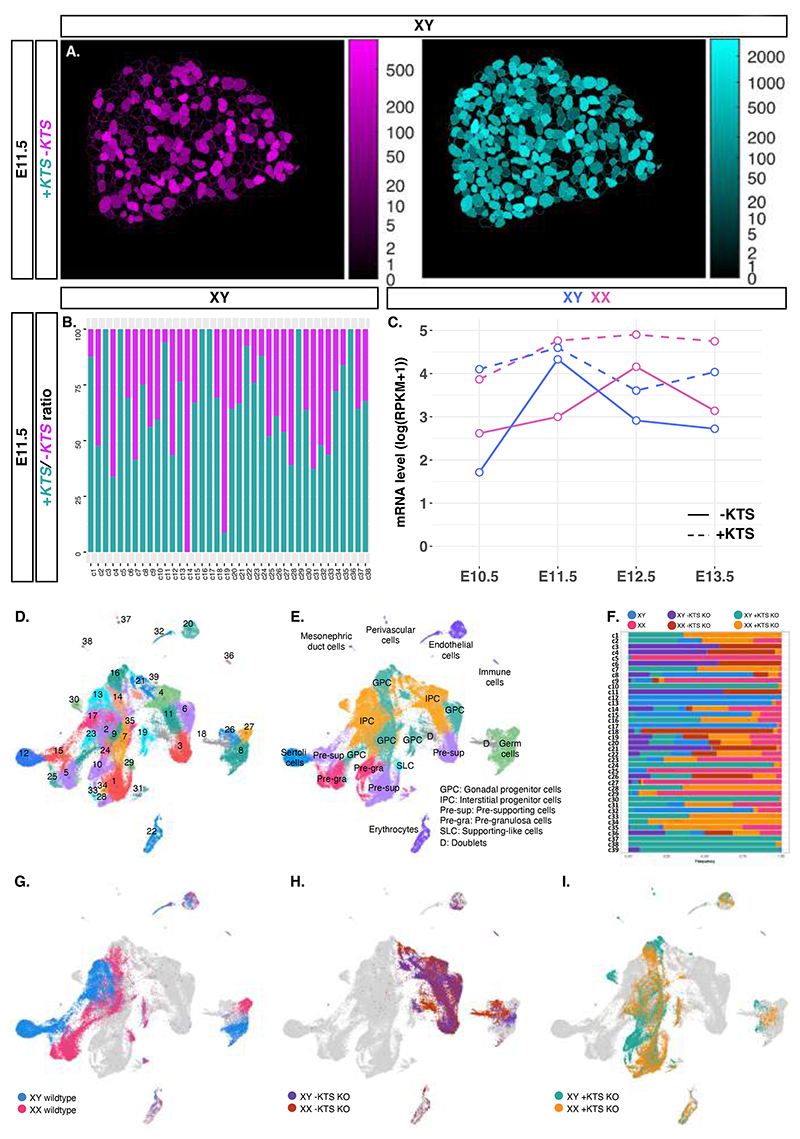
Dynamic distribution of *+KTS* and *-KTS* transcripts and single-cell transcriptomic analysis of *-KTS KO* and *+KTS KO* during early mouse gonad development. **(A)** Representative area distribution of *-KTS* (magenta) and *+KTS* (cyan) transcripts from Basescope in situ hybridizations on XY gonad sections at E11.5 (21ts (tail somites)) in μm^2^ per nucleus measured using DicHysto protocol. Data from both gonads are representative of biological and technical duplicates. **(B)** *-KTS* and *+KTS* mRNA ratio in E11.5 (21ts) XY wildtype individual cells. **(C)** *+KTS* and *-KTS* transcript levels in single-cell transcriptomic dataset of differentiating supporting cells. **(D)** UMAP projection of the 75,360 cells colored by clusters or **(E)** by associated cell types. **(F)** Association of cell clusters with genotypes. **(G-I)** UMAP projection by genotypes with XY (blue) and XX wildtype (pink) **(G)**, XY (purple) and XX *-KTS KO* (*-KTS^-^/-KTS^-^*) (brown) **(H)**, and XY (green) and XX *+KTS KO* (*+KTS^-^/+KTS^-^*) gonads (orange) **(I)**.

To determine the distribution of WT1 splice variants in E11.5 XY mouse gonads, we carried out BaseScope in situ hybridizations. Scoring revealed cellular heterogeneity, with cells containing variable amounts of *+KTS* or *-KTS* transcripts (Fig. 1A, Fig. S1A). This observation was confirmed by single-cell RNA sequencing analysis of the splice junction reads obtained from sorted cells dissected from E11.5 mouse gonads (Fig. 1B). Next, we examined single-cell transcriptomic data of the supporting cell lineage in both sexes from E10.5 to E13.5 (8) (Fig. 1C, Fig. S1B). Although +*KTS* exhibits similar mRNA levels between XY and XX gonads at E10.5 and E11.5, *-KTS* transcripts are detected in greater amounts in XY gonads at E11.5 before increasing in XX gonads at E12.5, timepoints that coincide respectively with Sertoli and pre-granulosa cell differentiation.

### -KTS is required for the differentiation of the supporting cells

**Fig. 2:**
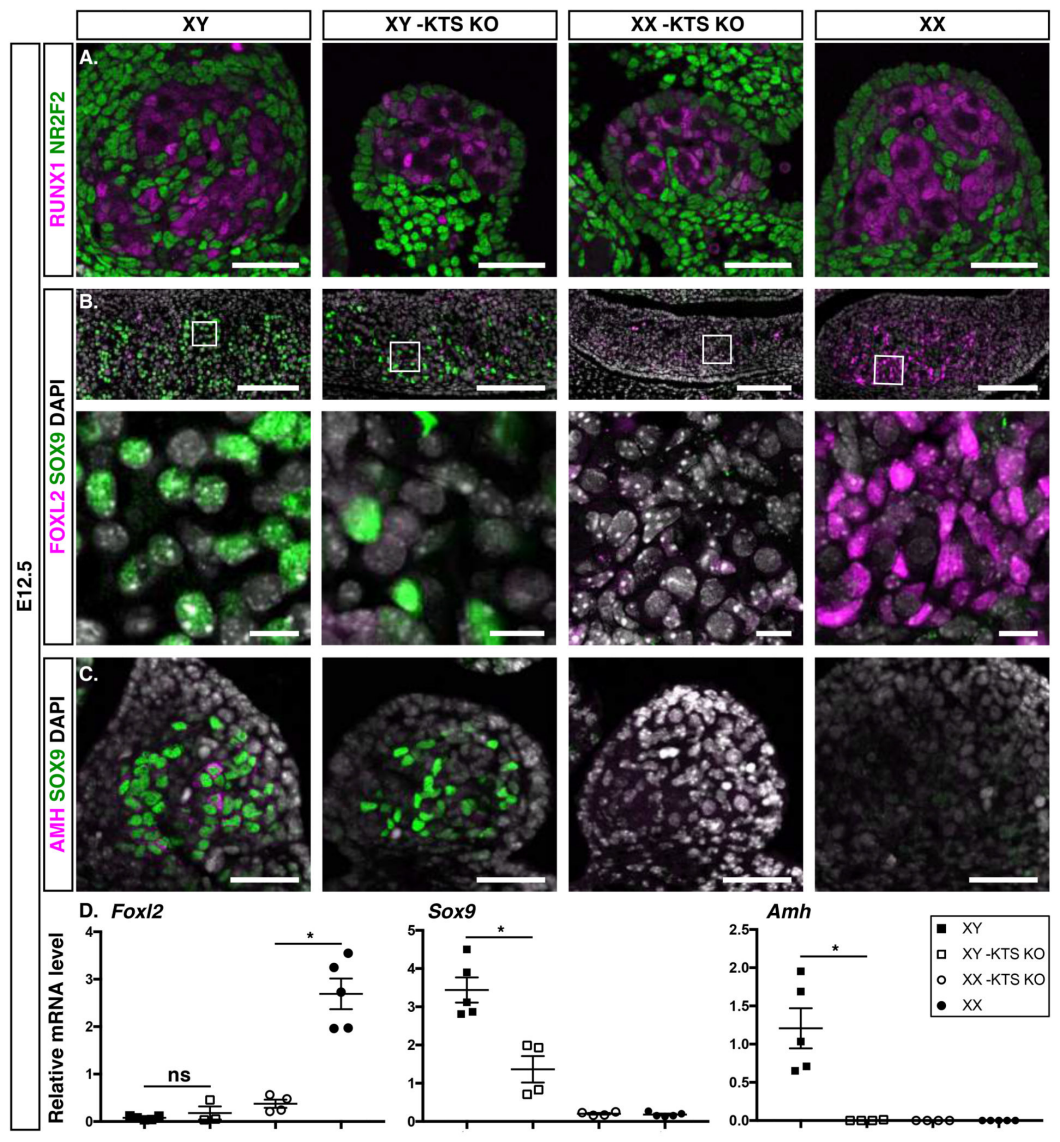
*-KTS* is necessary for sex differentiation of the supporting cells. Immunodetection of **(A)** the pre-supporting cell marker RUNX1 (magenta) and the progenitor marker NR2F2 (green) at E12.5 (scale bars: 50 μm), **(B)** the Sertoli cell marker SOX9 (green) and pre-granulosa cell marker FOXL2 (magenta) (scale bars: 100 μm, 10 μm), and **(C)** SOX9 (green) and AMH (magenta) (scale bars: 50 μm) in the indicated genotypes. **(A-C)** Data are representative of triplicate biological replicates. Nuclei labelled with DAPI are shown in white. (**D)** Quantification of *Foxl2*, *Sox9*, and *Amh* transcripts after normalization to *Gapdh* by RT-qPCR. Data are shown as means ± SEM. *-KTS KO: -KTS^-^/-KTS^-^*.

To address the contribution of *-KTS* to sex determination, we revisited the mouse model of *-KTS* ablation (*-KTS^-^/-KTS^-^* denoted *-KTS KO*) that results in gonadal dysgenesis (20) (Fig. S2A-B). We performed single-cell transcriptome profiling of wildtype and mutant gonadal cells collected around E12.0 (Fig. 1D-I, Table S1). Cells were projected in a 2D-space using UMAP and partitioned into 39 clusters (Fig. 1D). Cluster annotation identified Sertoli and pre-granulosa cells in the controls based on the expression of known markers (Fig. 1E-G, Fig. S3A-B), however these clusters were not present in *-KTS KO* gonads (Fig. 1E-H, Fig. S3B). Nevertheless, pre-supporting cells were observed in *-KTS* mutants of both sexes, as revealed by the expression of *Runxl(*mRNA*)* /RUNX1(protein) at E12.0 (Fig. S3B-cluster3) and at E12.5 (Fig. 2A, Fig. S4). XY *-KTS*-deficient gonads exhibited a few scattered cells expressing SOX9, contrasting with the widespread SOX9-positive Sertoli cells forming nascent testis cords in XY controls (Fig. 2B). Furthermore, XY *-KTS*-deficient gonads were devoid of *Amh*/AMH expression (Fig. 2C-D, Fig. S5A-B) and instead abnormally maintained *Rspo1* and SRY at E12.5 and until birth (Fig. S2C-E, Fig. S5C-E). Together, our results indicate that the pre-supporting cells did not differentiate as bona fide Sertoli cells in absence of -KTS. Despite the significantly reduced expression of *Sox9 (p*-value = 0.0035), the XY *-KTS KO* pre-supporting cells failed to differentiate into pre-granulosa cells, as evidenced by the almost complete absence of *Foxl2/FOXL2* expression at E12.5 (Fig. 2B, 2D, Fig. S3). Similarly, *Foxl2/FOXL2* expression was strongly reduced in XX *-KTS KO* gonads, further supporting the importance of -KTS for the differentiation of the pre-granulosa lineage and the activation of the female program (Fig. 2B, 2D, Fig. S3). Around birth, the expression of *Sox9/*SOX9 and *Foxl2/*FOXL2 remained low in -*KTS*-deficient gonads of both sexes, and SOX9/FOXL2 double-positive cells were detected (Fig. S5F-H), indicating poor differentiation of the supporting lineage. Together these data demonstrate that *-KTS* is dispensable for the specification of pre-supporting cells but is necessary to stabilize Sertoli cell differentiation and essential to initiate pre-granulosa cell differentiation.

### Absence of +KTS triggers an increase of -KTS amounts

**Fig. 3:**
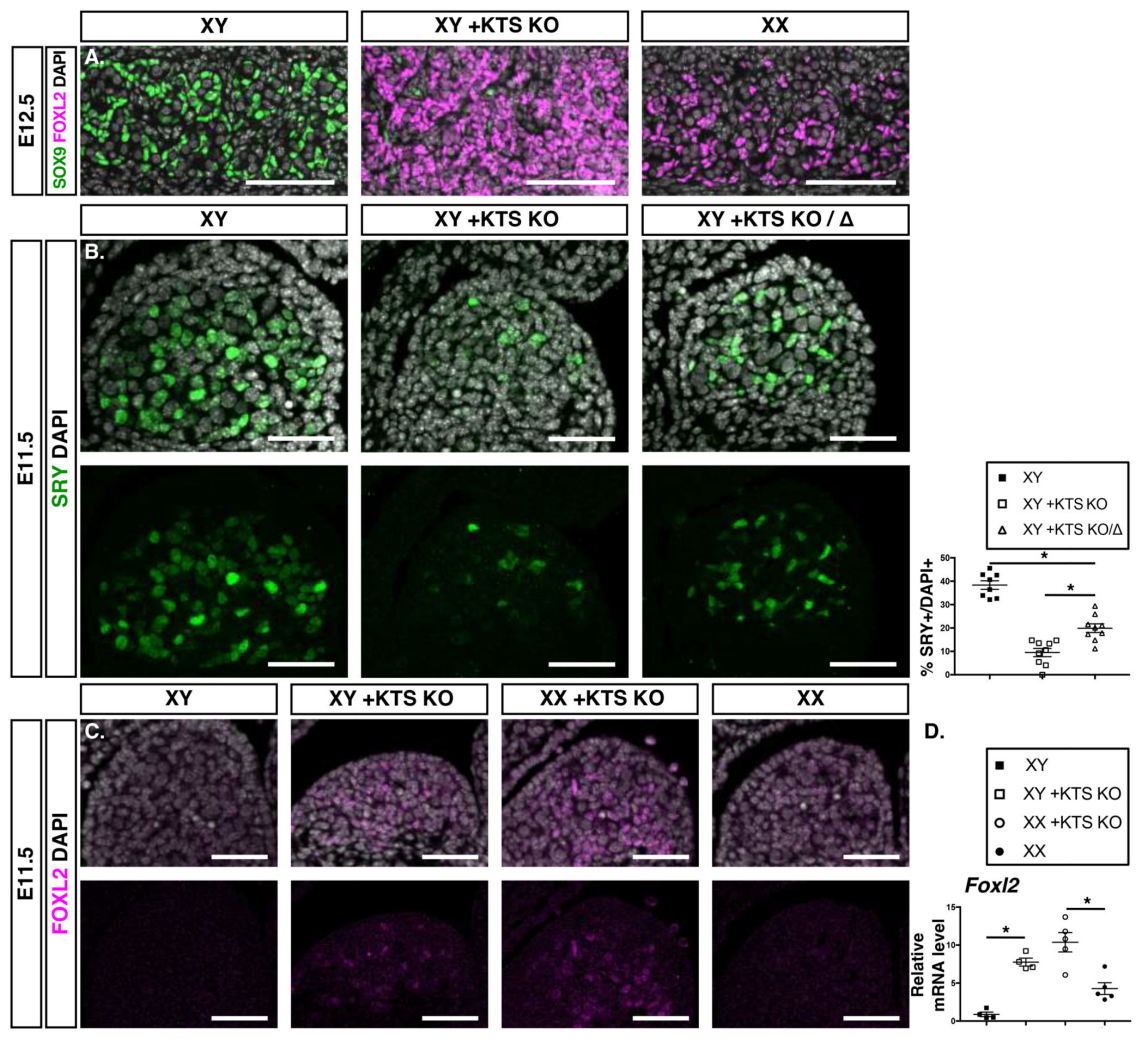
Early pre-granulosa cell differentiation occurs in XY and XX *+KTS KO* gonads. **(A)** Immunofluorescence of the Sertoli cell marker SOX9 (green) and the pre-granulosa cell marker FOXL2 (magenta) in indicated genotypes at E12.5 (n=4). Scale bars: 100 μm. **(B)** Immunostaining of SRY (green) at E11.5 (21±1ts) in XY, XY *+KTS KO* (*+KTS^-^/+KTS^-^*), and XY compound heterozygotes *(+KTS KO/Δ: +KTS/Wt1-)*. Scale bars: 50 μm. Quantification of SRY+ cells normalized to DAPI+ cells labeled in white in the upper panel. n=4, 2 sections/embryo. Data are shown as means ± SEM. **(C)** Immunodetection of the pre-granulosa cell marker FOXL2 in indicated genotypes of triplicate biological replicates at 20-21ts. Scale bars: 50 μm. **(D)** Relative mRNA expression of *Foxl2* normalized to *Gapdh* at 20-21ts. Data are shown as means ± SEM.

In patients with Frasier syndrome, heterozygous mutations in WT1 prevent the production of *+KTS*, resulting in higher amounts of *-KTS* variants (18, 19). Given the role of *-KTS* in ovarian determination, we investigated the contribution of this increase to sex reversal in the *+KTS KO* (*+KTS-/+KTS–*) mouse model (20). At E12.5, XY *+KTS* mutant gonads were enriched for RUNX1 and FOXL2-positive pre-granulosa cells and contained rare Sertoli cells, as expected for male-to- female sex reversal (Fig. 3A, Fig. S6A). Next, we verified that *-KTS* transcripts were twice as abundant in *+KTS KO* gonads compared to controls (Fig. S6B). In addition, total WT1 protein levels were similar in XY *+KTS KO* and control gonads, confirming that absence of +KTS is compensated with an increase of -KTS isoforms (Fig. S6C).

### Precocious pre-granulosa cell differentiation prevents *Sry* activation in the mouse Frasier model

Sex reversal in *+KTS KO* embryos is caused by a failure of *Sry* activation (20, 21). To determine if this was due to the lack of *+KTS* or an increase in *-KTS* variants, we compared the number of SRY-positive cells in +*KTS KO* and *+KTS KO/D* compound embryos, both of which lack alleles encoding *+KTS* and contain two and one allele encoding *-KTS*, respectively. The number of SRY expressing cells was higher in *+KTS KO/D* compared to XY *+KTS KO* gonads, indicating that *Sry* expression does not require +KTS but is antagonized by the higher level of -KTS (Fig. 3B). Furthermore, *Rspo1* was abundant in XY *+KTS KO* gonads at E11.5, a stage when it is downregulated in XY gonads, and *Foxl2*/FOXL2 expression was markedly elevated in XY and XX *+KTS KO* gonads (Fig. 3C-D, Fig. S6E). This suggests precocious pre-granulosa cell differentiation irrespective of the genetic sex.

### -KTS is sufficient to induce ovarian development

**Fig. 4:**
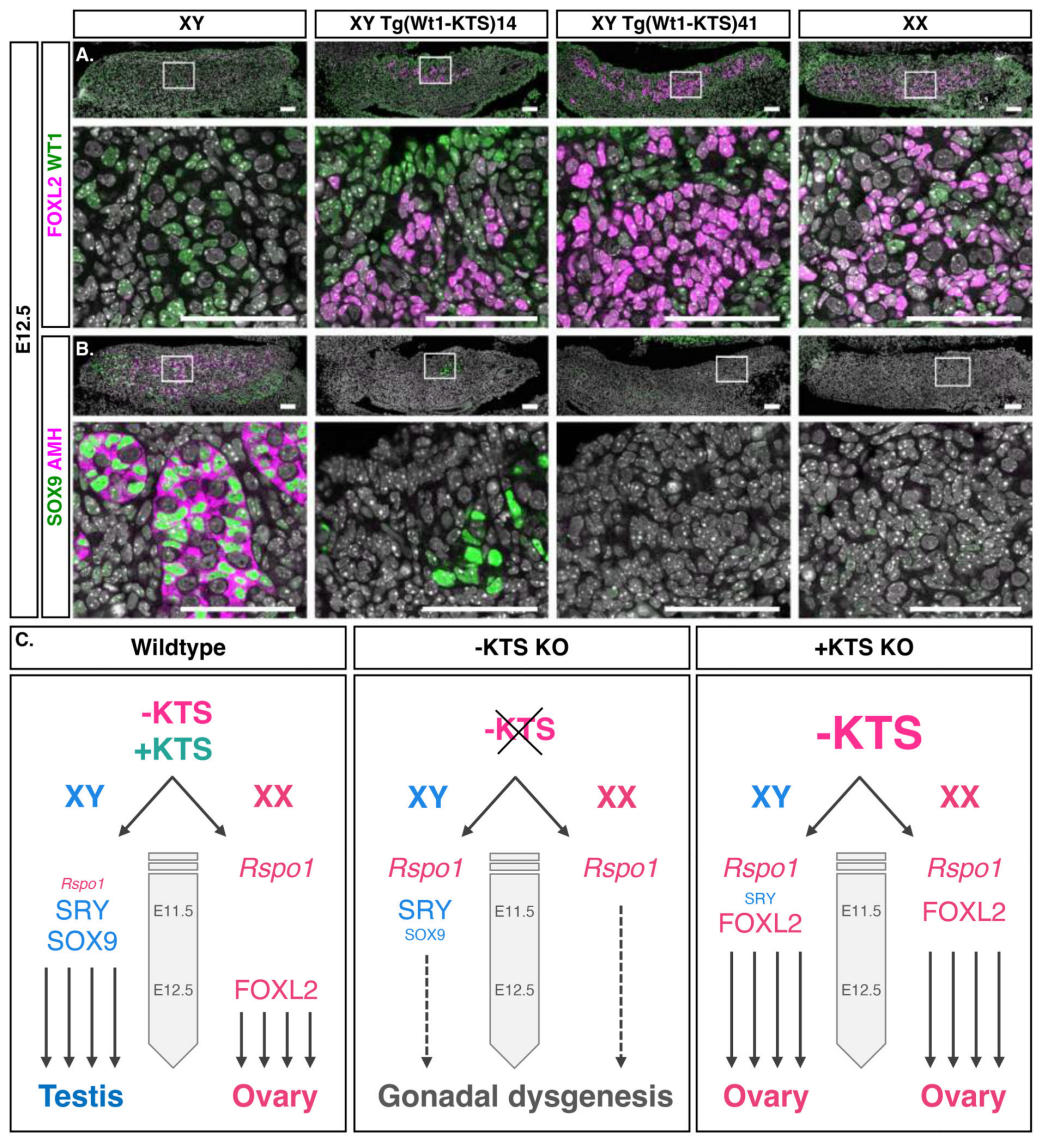
*-KTS* induces pre-granulosa cell differentiation in XY transgenic gonads. (**A**) Immunofluorescence of the pre-granulosa cell marker FOXL2 (magenta) and WT1 (green) in the indicated genotypes at E12.5. Scale bars: 50 μm. **(B)** Immunostaining of the Sertoli cell markers SOX9 (green) and AMH (magenta) in indicated genotypes. Scale bars: 50 μm. **(C)** Model of supporting cell differentiation in wild-type and *KTS* mutant gonads: absence of *-KTS* in *-KTS KO* gonads promotes the maintenance of *Rspol* transcripts and impairs SOX9 and FOXL2 expression leading to gonadal dysgenesis. Increasing -KTS in *+KTS KO* gonads results in ovarian differentiation in both genetic sexes.

Single-cell transcriptome profiling of XY and XX *+KTS KO* gonads at E12.0 (Fig. 1D-F, 1I, Fig. S3B) identified two pre-granulosa cell clusters (c10, c33) distinct from those found in XX controls (c5, c25) and from XY Sertoli cells (c12). Further comparison of transcriptomes of these clusters confirmed that E12.0 *+KTS KO* cells are transcriptionally related to pre-granulosa cells (Fig. S7). Next, we used comparative analysis to identify genes that are activated or repressed by -KTS in the context of female sex determination (Fig. S8). In XX *-KTS KO* pre-supporting cells, the expression of 319 genes was significantly deregulated (FDR adjusted p-value ≤ 0.05, Data S4). *Pdgfa* and *Tcf21*, reported to be targets of WT1 in other organs (22, 23), were down-regulated, whereas *Igf2*, a direct target of WT1 (24), and genes highly expressed in bipotent pre-supporting cells including *Sprr2d* (25), *Wnt6* (26), *Nr0b1* (27), were up-regulated in XX *-KTS KO* pre-supporting cells and down-regulated in XX *+KTS KO* pre-granulosa cells, suggesting that they are repressed by -KTS during sex determination. Altogether our data suggested that increased –KTS rather than loss of +KTS was responsible for XY sex reversal in *+KTS KO* model. To test whether -KTS was sufficient to induce ovarian differentiation in a XY wildtype gonad, we performed transient additive transgenesis using a BAC construct covering the *Wt1* locus, in which we introduced the classical Frasier mutation in intron 9 (interference with +KTS production). Strikingly, 3 out of 4 XY transgenic animals showed the presence of FOXL2 positive cells indicating that -KTS promotes differentiation of pre-granulosa cells in XY gonads (Fig. 4A-B, Fig. S9A-B). Moreover, RT-qPCR analysis of genotypes producing different levels of -KTS suggested that -*KTS* must reach a threshold to robustly activate *Foxl2* expression (Fig. S9C).

In summary, we can conclude that the altered expression of *+KTS* caused by mutations in the donor splice site in intron 9 of *Wt1* promotes an increase of the amount of -KTS, which in turn prematurely activates ovarian differentiation, prevents *Sry* up-regulation, and impairs testis development (Fig. 4C). -KTS thus represents a key actor in gonad development that is required to initiate ovarian development.

## Discussion

Here, we provide evidence that sex determination does not only depend on the up-regulation of the sex-determining factors, *Sry* and *-KTS* for male and female fates respectively, but also on their timing (28-30). This is an important concept as *-KTS* is an autosomal factor expressed in both XY and XX gonads. In wildtype mice, *Sry* acts before *-KTS*, thus securing testis development in XY gonads. If *Sry* expression is impaired or delayed, or if *-KTS* is up-regulated prematurely, such as in the Frasier syndrome model *(+KTS KO*), the pre-granulosa cell differentiation is accelerated leading to male-to-female sex reversal. After the peak of SRY action (30), -KTS becomes necessary to maintain Sertoli cell differentiation in XY embryos and to initiate pre-granulosa cell differentiation in XX embryos (Fig. 4C). While differences in timing and dynamics of sex determination make a direct comparison between mouse and human data difficult, the sex reversal phenotype in mice carrying intron 9 mutations suggests this is a good mouse model for the human Frasier syndrome. Our data thus indicate that increased expression of -KTS rather than loss (or reduction) of +KTS are the primary cause of sex reversal in Frasier syndrome. Interestingly, a change of *+KTS/-KTS* ratio in favor of *-KTS* operates when the eggs of *Chelydra serpentina*, a turtle with temperature-dependent sex determination, are shifted from a male to a femaleproducing temperature (31). This suggests that the -KTS isoform of WT1 is also involved in ovarian determination outside of the mammalian class.

## Supplementary Material

Data S1

Data S2

Data S3

Data S4

Supplementary Material

## Data Availability

All data are available in the manuscript or the supplementary materials. The scRNA-seq datasets are available at NCBI Gene Expression Omnibus GEO: GSE207097 and in articles cited in the paper. Programs developed are available on Zenodo (32–34).

## One-Sentence Summary

The -KTS splice variant of WT1 has a dual function in mediating gonadal sex determination
