# Supplementary material for: The -KTS splice variant of WT1 is essential for ovarian determination in mice
Source: Science. Author manuscript; Available in PMC 2023 Nov 16. (PMC7615308; doi:10.1126/science.add8831)
Supplement: Supplementary Material [file EMS190512-supplement-Supplementary_Material.pdf]

Science
AAAS

Supplementary Materials for

The *-KTS* splice variant of WT1 is essential for ovarian determination in mice

Elodie P Gregoire[1], Marie-Cécile De Cian[1†], Roberta Migale[2†], Aitana Perea-Gomez[1], Sébastien Schaub[3], Natividad Bellido-Carreras[1], Isabelle Stévant[4,5], Chloé Mayère[4,5], Yasmine Neirijnck[1], Agnès Loubat[1], Paul Rivaud[6], Miriam Llorian Sopena[2], Simon Lachambre[7], Margot M. Linssen[8], Peter Hohenstein[8], Robin Lovell-Badge[2], Serge Nef[4,5], Frédéric Chalmel[6], Andreas Schedl[1], Marie-Christine Chaboissier[1]*.

Correspondence to: Marie-Christine.CHABOISSIER@univ-cotedazur.fr.

This PDF file includes:

Materials and Methods
Figs. S1 to S9
Tables S1 to S3
References (35–49)

Other Supplementary Materials for this manuscript include the following:

Data S1 to S4

Materials and Methods

Mouse strains and genotyping

All experiments described herein were conducted in compliance with the relevant institutional and French animal welfare laws and policies. Mouse lines were kept on a mixed background B6CBAF1/JRj. The *Wt1*^{tm1Jae} (referred to as Δ), KTS (*-KTS*$^{-}$/*-KTS*$^{-}$ referred to as *-KTS KO*) and Frasier (*+KTS*$^{-}$/*+KTS*$^{-}$ referred to as *+KTS KO*) lines have been described previously in (*15*, *20*). Heterozygous *+KTS*$^{-}$/+ were mated to Δ/+ mice to obtain compound heterozygous referred to as *+KTS KO*/Δ embryos. The day of the presence of a vaginal plug was marked embryonic day 0.5 (E0.5). Embryos collected at E11.5-12.0 were staged by counting the number of tail somites (ts) with 18 ts corresponding to E11.5 (*35*). The day of delivery was defined as post-natal day 0 (P0). The genotyping was performed on lysates of tail tips or ear biopsies as described previously in (*20*, *36*). The primers are listed in Table S2.

BAC Wt1-KTS transgenic line: BAC *Wt1*, RP24-67H19 (*37*) was obtained from the Children's Hospital Oakland Research Institute (CHORI, USA) and modified by recombineering techniques performed by Gen-H GmbH (Genetic Engineering Heidelberg) by insertion of the point mutation T->C in the second splice donor site at position +2 in intron 9 as described in (*20*). This promotes the expression of *-KTS* and prevents the synthesis of *+KTS* variants. The BAC was digested by NotI to obtain linear DNA and dialyzed against 10 mM Tris:HCl pH7.4, 0.1 mM EDTA to a final concentration of 2 ng/µl (Fig. S9A). Microinjection was performed using fertilized oocytes as described in (*37*). The embryos were collected at E12.5 from the fosters, genotyped as described in (*20*), and gonads were processed for immunological analyses. Four XY transgenic embryos *Tg*(RP24-67H19; *Wt1-KTS*) were obtained with different degrees of mosaicism. The transgenic embryos denoted *Tg(Wt1-KTS)14* and *Tg(Wt1-KTS)41* are shown in Fig. 4A-B, Fig. S9B.

In situ hybridization

Tissues were fixed overnight at room temperature with 4% paraformaldehyde, processed for paraffin embedding, and sectioned at 5 µm thick. *Rspo1* mRNA were detected using the RNAscope technology (479591 probe) and *-KTS* and *+KTS* variants using the Basescope Duplex Detection Kit (715291-C2 and 715281 probes, respectively). The protocol was performed according to Advanced Cell's instructions using the chromogenic Fast Red or Fast Green dye. Slides were counterstained with Hoechst, and images were obtained on microscopes (LSM780, AxioObservera and Axiovert 200M, Carl Zeiss).

Image analysis of Basescope in situ hybridization

The image acquisition was performed with a Axiovert 200M (Zeiss) with 10x/0.3 objective equipped with an Andor Neo camera. From this setup, an analysis was carried out in 4 steps, called DICHisto. First, we developed in Metamorph (Molecular Devices, Sunnyvale, CA) a program to acquire sequentially, in DIC, the wavelength range 440/60, 525/50, and 605/70 corresponding to color camera RGB (but without Bayer demosaicing artefact). After an auto-calibration step (optimizing exposures for global white balance), the process could be inserted in multi-dimensional acquisition (Z, time, mosaic) and combined with fluorescence. Second, we developed in Matlab (The Mathworks, Natick US), a program to quantify the amount of Basescope staining of *+KTS* and *-KTS* staining from raw images. We corrected inhomogeneous illumination with a local white balance. After converting in HSL, we extracted “FHisto” signals corresponding to the saturation value filtered based on hue for *+KTS* and *-KTS* (and to a lesser extent the luminance). The luminance channel corresponds to the monochromatic DIC image. Then this generated a

multi-channel stack including FHistos, DIC, and fluorescence channels (DAPI and others if any). Third, we developed a set of ImageJ macros that is designed for interactive whiteboard. We drew manually the nuclei of the gonad (a second category has been defined for indiscernible nuclei). Then the program quantified the statistics per image including the area of *+KTS* and *-KTS* per nucleus and in the cytoplasm. And fourth, we summarized all statistical parameters developing another Matlab program. All programs with test images are available (*32*).

Immunological analyses

Gonad samples were fixed, embedded, and sectioned as described for in situ hybridization. Sections were rehydrated, boiled in a pressure cooker for 2 min with Antigen Unmasking Solution (Vector laboratories), and blocked in PBS solution containing 10% normal donkey serum and 3% BSA. All antibodies were applied overnight at 4 °C at the concentrations listed in Table S3. Alexa-conjugated secondary antibodies were diluted 1:200 and applied at room temperature for 1 h. Slides were counterstained with DAPI diluted in the mounting medium (Vectashield, Vector Laboratories). The images were generated with a motorized Axio Imager Z1 microscope (Zeiss) or with confocal laser scanning microscope (LSM780, Carl Zeiss). For wholemount immunofluorescence, the protocol was performed as described in (*38*). The 3D reconstitution of SOX9 staining have been processed with Imaris 9.1 Bitplane.

Image post-treatments

Image post-treatments were performed on Z-stack maximum intensity projection using a homemade semi-automated macro on ImageJ software (National Institutes of Health) available on Zenodo (*33*, *34*).

Quantification of RUNX1 and NR2F2 positive cells.

Gonadal Region Of Interest (ROI) was drawn manually, and nuclei were segmented upon DAPI staining using Versatile (fluorescent nuclei) model from Stardist Deep Learning plugin (*39*). Thresholds defining RUNX1 of NR2F2 cell positiveness were adjusted and applied to each nuclei area defined by Stardist to obtain the number of cells positive for RUNX1, NR2F2, double positive, or double negative (*33*) (Fig. S4A). For each genotype, 2 gonads of 3 or 4 biological replicates were analyzed. Statistical significance was determined with Student's unpaired two-tailed t-test (GraphPad Prism v.7.0b). * indicates p-value ≤ 0.05; ns indicates p-value > 0.05.

Quantification of WT1 signal intensity

Gonadal WT1 signal was determined as the WT1 signal colocalized with GATA4 immunostaining (GATA4-positive cells allow manual surrounding of the gonad) and stored in region of interest (ROI) manager as gonad ROI (Fig. S6D). Automatic mean threshold adjustment was performed on WT1 signal intensity and small object rejected using analyze particles filter (10-infinity). The mean signal intensity within the gonad ROI was quantified after threshold adjustment. The average takes into account the number of selected pixels and therefore the area. The comparison between each set of experiments was performed by adjusting the WT1 signal intensity of the XY control gonad in each set to the same level (*34*). For each genotype, 2 sections and 4 biological replicates. Statistical significance was determined with Student's unpaired two-tailed t-test (GraphPad Prism v.7.0b). ns indicates p-value > 0.05.

Image processing

The DAPI/Hoechst staining marked the nuclei and was adjusted to visualize the tissues and may vary between samples. However, for the immunostaining analysis, the exposure time of the

acquisition of the fluorescent signal was identical in the same experiment to allow comparison between wild type and mutants. Images were assembled using the open-source software platform OMERO (https://www.openmicroscopy.org/omero/).

Quantitative PCR analysis

Individual gonads were dissected from the mesonephros in PBS and immediately frozen in liquid nitrogen and kept at -80 °C. RNA was extracted by the RNeasy Micro Kit (Qiagen) and reverse transcribed by the MMLV reverse transcriptase (Promega). The cDNA was used as a template for quantitative PCR analysis using the SYBR Green I Master (Roche) and a LightCycler 480 System (Roche). Primer sequences are listed in Table S2. All biological replicates of different genotypes (n=3-5) are representative of independent duplicate technical replicates. Statistical significance was determined with Student's unpaired two-tailed t-test (GraphPad Prism v.7.0b). * indicates p-value ≤ 0.05; ns indicates p-value > 0.05.

Single-cell RNA transcriptomic analysis of XY gonads collected at E11.5

XY wild-type gonads were dissected from C57BL/6J embryos at E11.5 (21ts). Tissue was digested in 450 µL of 0.05% Trypsin and 50 µL of 2.5% collagenase and incubated for 8 minutes at 37°C. Digestion reaction was quenched with 200 µL of PBS-FBS 3%, and genital ridges were disaggregated by gently pipetting. Cells were passed through a 30 µM cell strainer into a tube to obtain single cell suspension. MoFlo XDP fluorescence-activated cell sorting (FACS) was used to isolate single cells into a chilled 96-well plate pre-loaded with 12.5 µL of CDS sorting solution (SMARTseq HT Takara Bio cat. # 634437).
Following cDNA synthesis and amplification with SMARTseq HT kit, cDNA quality was assessed by Tapestation. A total of 49 cells out of 60 passed the quality checks, and indexed libraries were prepared using Nextera XT DNA Sample Preparation kit and multiplexed.
RNA-Seq libraries were sequenced as 100 bp paired-end reads using the Illumina HiSEQ4000 platform at a depth of 10M reads per cell.
Reads were aligned to the Ensembl mouse reference GRm38 (release 89) using STAR (v2.5.2a) with default parameters (*40*). Gene level quantification was performed using RSEM (v1.3.0) (*41*), and junction counts corresponding to the region of interest were extracted using BEDTools (v 2.27.1) (*42*). To quantify the *KTS* splice variants, we used uniquely mapped reads and calculated the percent spliced in (PSI) as the fraction of reads mapping to the +*KTS* or -*KTS* variants divided by the sum of both.

Single-cell RNA transcriptomic analysis of wildtype, *-KTS KO,* and *+KTS KO* gonads

E12.0 (23±3ts) gonads from wild-type, -*KTS KO,* and +*KTS KO* mice (Table S1) were enzymatically dissociated for 10 min at 37°C using trypsin-EDTA 0.05% (Gibco, Fisher Scientific, Hampton, NH). After centrifugation, single cells were loaded on a 10× Chromium instrument (10× Genomics, Pleasanton, CA). Single-cell RNA sequencing libraries were prepared using the Chromium Single Cell 3′, version 3, reagent kit (10× Genomics), according to the manufacturer's protocol. Each genotype was performed in two independent replicates. The library quantification and quality assessment were performed using an Agilent Bioanalyzer 2100 with a high sensitivity DNA chip (Agilent Technologies, Santa Clara, CA). The libraries were diluted, pooled, and sequenced using an Illumina HiSeq4000 using paired-end 29 × 101 bp as the sequencing mode.

Fastq files were processed with CellRanger (v6.0), and the resulting count matrices were aggregated with the Read10X function implemented in Seurat (v4.0.1) (*43*). Doublets were filtered

out independently in each individual matrix by using the DoubletFinder R package (v.2.0.2) (*44*) We next filtered out low-quality cells by retaining cells that expressed ≥1000 genes and had mitochondrial content ≤10%. Data were normalized using the NormalizeData and the SCT function implemented into Seurat. The top-3000 most varying genes were used to perform a principal component analysis with the RunPCA function implemented in Seurat. Cells were then clustered by using Seurat graph-based clustering (FindNeighbors and FindClusters functions) on the top-50 principal components, with default parameters. Finally, we used the Uniform Manifold Approximation and Projection (UMAP) method (RunUMAP function) to project cells in a 2D space. Cell clusters were annotated using a set of known marker genes. The FindMarkers function implemented in Seurat was used to identify significantly differentially expressed genes (DEGs) (FDR adjusted p-value ≤ 0.05) between wildtype pre-granulosa cells (belonging to cell clusters c5 and c25) to XX and XY *+KTS KO* pre-granulosa cells (c10, c33), and between wildtype Sertoli cells (c12) to XX and XY *+KTS KO* pre-granulosa cells (c10, c33). The resulting set of DEGs was further partitioned into 9 clusters by using the kmeans algorithm graphically represented as a heatmap with color-coding as shown in Fig. S7 using the pheatmap package implemented in R. Gene ontology (GO) and pathway enrichment analysis was conducted for each gene expression cluster using the AMEN suite of tools (*45*) with an BH-adjusted p value of ≤ 0.05. The FindAllMarkers function implemented in Seurat was used to identify significantly differentially expressed genes (DEGs) (FDR adjusted p-value ≤ 0.05) between cell clusters in Fig. S8.

Quantification of alternative splice variants +*KTS* and -*KTS* in single-cell transcriptomic dataset of supporting cell lineage

To calculate the usage of the +*KTS* and -*KTS* transcript along gonadal development in both sexes, we used the single-cell RNA-sequencing data described in (*8*, *46*) (GEO GSE97519 and GSE119766, respectively). Briefly, the data were obtained from *Nr5a1* expressing cells of embryonic gonads of both sexes using the C1 autoprep system from Fluidigm at different time points of the gonadal development (E10.5, E11.5, E12.5, and E13.5). Their transcriptomes were mapped on the mouse reference genome (GRCm38.p3) and the genome annotation from GENCODE (version M4, modified to integrate the GFP transgene). Cells were then clustered according to their transcriptomic profiles using the HCPC clustering method from FactoMineR R package, and the cellular identity of each cluster was assessed by the co-expression of known gonadal cell marker genes. Cell lineage reconstruction was performed using Slingshot and diffusion map from the Destiny R package to model the trajectory of the cell differentiation.

To quantify the -*KTS* transcripts in each cell, we postulated that the exon 9 of *Wt1* is a constitutive exon, that is always present in the *Wt1* transcript. The quantity of +*KTS* transcripts was calculated by comparing the proportion of reads covering the *KTS* sequence compared to the rest of exon 9. To proceed, we extracted the reads of the *Wt1* gene locus from the BAM files using Samtools and only kept the uniquely mapped and properly paired reads. For each cell we calculated the mean depth of coverage (the number of times a nucleotide was read during sequencing) of the nucleotides of *KTS* and exon 9 of *Wt1* using the coverage option from Samtools. We selected the cells having at least 5 reads covering the *KTS* sequence and/or exon 9 to discard cells in which the exon quantification was too low and considered low confidence. Finally, we obtained the amount of +*KTS* and -*KTS* transcripts per cell by calculating the ratio of coverage of the *KTS* sequence compared to the exon 9. A cell with no coverage (no reads) on the *KTS* sequence was considered as devoid of +*KTS* (0%). Conversely, a cell having as much or more coverage on the *KTS* sequence than on exon 9 is considered having 100% of the +*KTS*. To estimate the expression of the respective +*KTS* and -*KTS* variants in each cell, we reported the percentage of +*KTS* and -*KTS* to the RPKM values previously calculated (*8*).

To follow the proportion of +*KTS* variants in the supporting cell lineage as they differentiated into either Sertoli or pre-granulosa cells, we selected the cells of the supporting cell lineage from their multipotent progenitor state at E10.5 until their differentiated state at E13.5, as previously defined (*8*). We calculated the mean percentage as well as the mean expression of +*KTS* and -*KTS* variants of the supporting cells at each stage. Plots were generated using R version 4.2 and the Ggplot2 library.

Figure S1

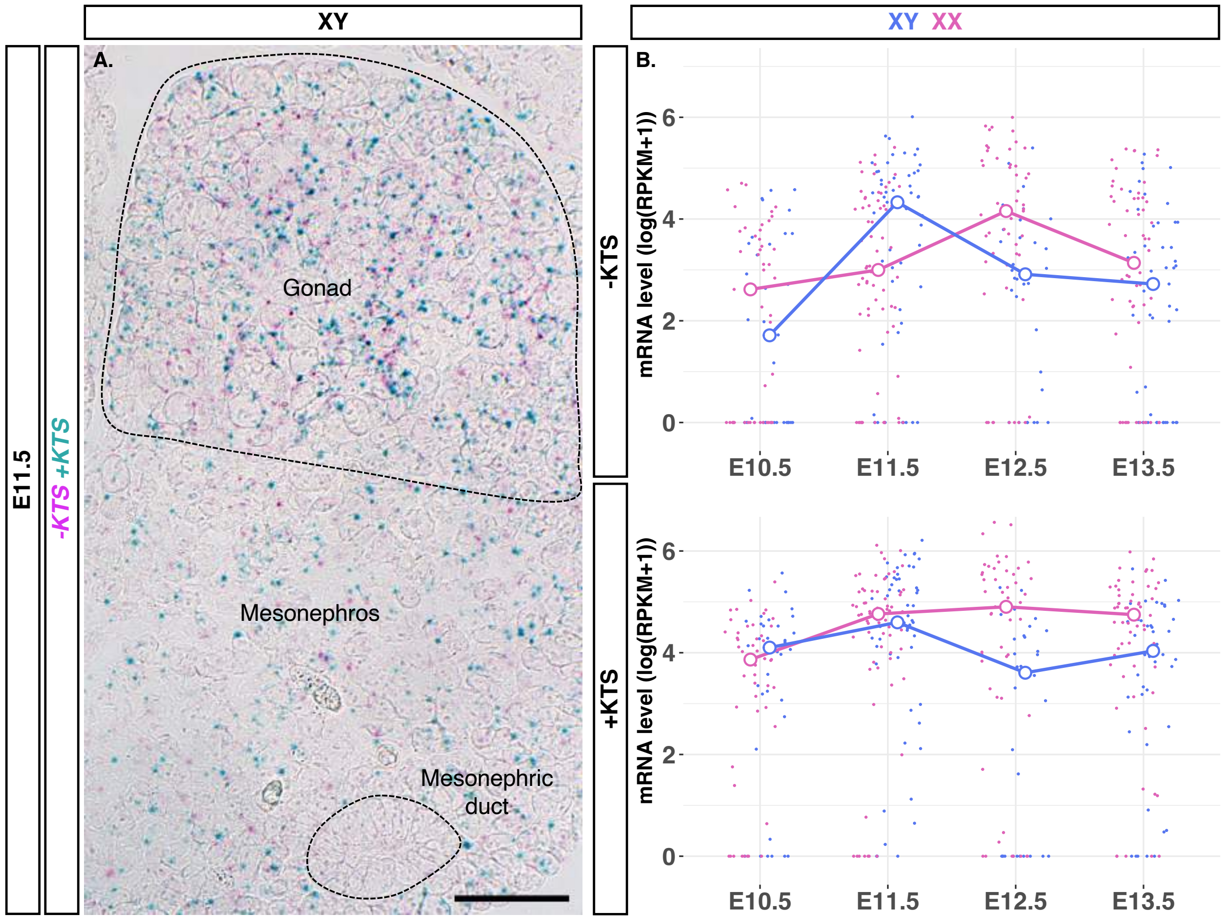

Fig. S1. Heterogeneity of +*KTS* and -*KTS* transcripts.
(A) BaseScope in situ hybridization of +*KTS* (cyan) and -*KTS* (magenta) on E11.5 (21-22ts) XY sections visualized by light microscopy. *Wt1* is not expressed in the mesonephric ducts. Scale bar: 50 µm. **(B)** Amounts of both transcript isoforms, +*KTS* and -*KTS* in single-cell RNA-seq dataset of differentiating supporting cells.

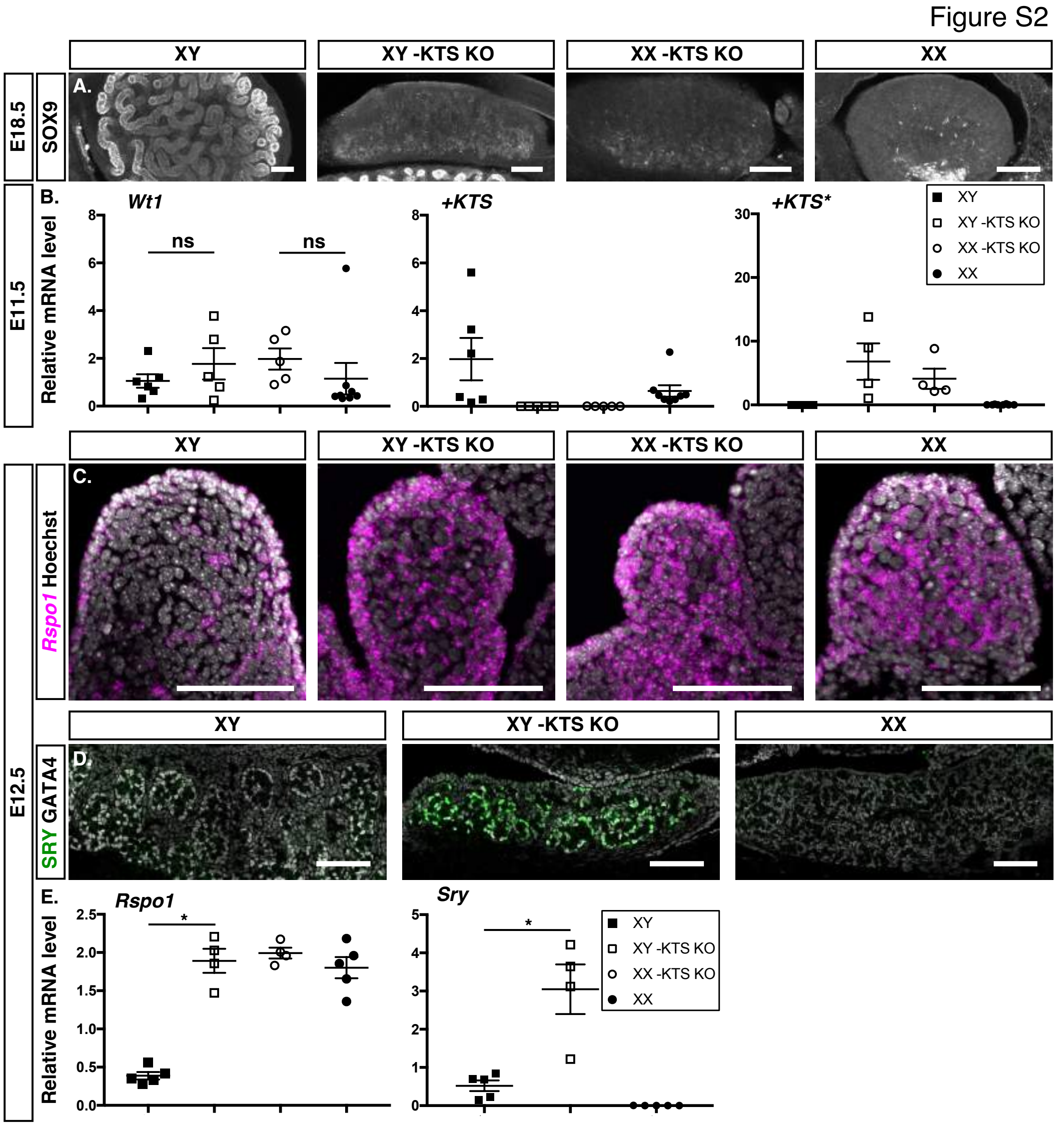

Fig. S2. Gonadal development is arrested in absence of -*KTS*.
(A) Immuno-localization of the Sertoli cell marker SOX9 in whole gonads using Imaris highlighting the absence of organized testis cords at E18.5 in -*KTS KO* gonads. Scale bars: 100 µm. **(B)** RT-qPCR analysis of *Wt1*, wildtype +*KTS* and mutated +*KTS** mRNA levels normalized to *Gapdh* indicating that absence of -*KTS* is compensated with an increase of +*KTS* variants leading to similar level of *Wt1* transcripts in indicated genotypes at E11.5. An asterisk is added to +KTS (+KTS*) when one of the primers used in the RT-qPCR contains the A to C and T to G mutations identical to the -*KTS KO* model. The primers corresponding to the wild-type sequence do not match the mutated sequence and vice versa. Data are shown as means ± SEM. **(C)** RNAscope in situ hybridizations of the progenitor or pre-granulosa cell marker *Rspo1* reveal the failure of *Rspo1* down-regulation in XY -*KTS KO* gonads at E12.5 (n=3 biological replicates). Scale bars: 100 µm. Nuclei labelled with Hoechst are shown in white. **(D)** SRY is maintained in XY -*KTS KO* gonads at E12.5 as evidenced by immunostaining (n=3 biological replicates). Scale bars: 100 µm. **(A, C-D)** Data are representative of triplicate biological replicates. **(E)** RT-qPCR analysis of *Rspo1* and *Sry* mRNA levels normalized to *Gapdh* in indicated genotypes at E12.5. Data are shown as means ± SEM.

Figure S3

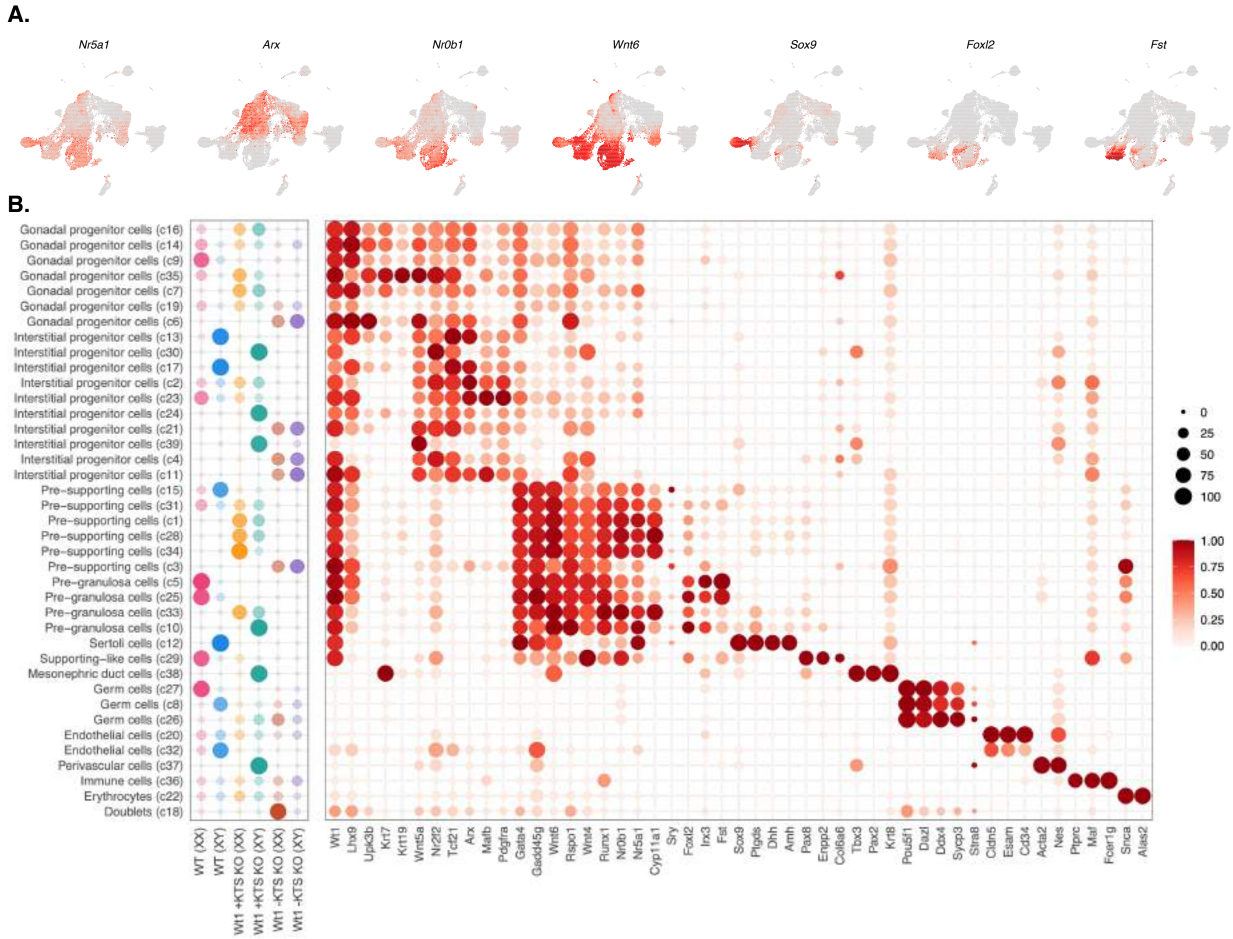

Fig. S3. Annotation of the clusters from single-cell transcriptomic analysis of gonads from *+KTS KO*, *-KTS KO,* and wild-type XY and XX embryos.
(A) UMAP projection of cells colored by expression (log normalized counts) of a marker of somatic cells *Nr5a1* (*47*), a marker of interstitial progenitors *Arx*, (*48*), a marker of pre-supporting cells *Nr0b1* (*49*), a marker of pre- and supporting cells *Wnt6*, a marker of the Sertoli lineage *Sox9*, two markers of the pre-granulosa cells *Foxl2* and *Fst* (*8*, *49*). **(B)** On the left-hand side: dot plot with the representation of the genotypes in the different clusters with XY control (blue), XX control (pink), XY *+KTS KO* (green), XX *+KTS KO* (yellow), XY *-KTS KO* (purple), XX *-KTS KO* (brown) cells. The dot size represents the prediction score (ranging from 0.0 to 1.0) of the cell cluster to be associated with a genotype based on the TransferData function implemented in Seurat. The color intensity indicates the percentage of cells of the cluster associated with a genotype. On the right-hand side: dot plot with the expression of selected markers (x axis) per cell cluster (y axis) indicated on the right-hand side of the dot plot. The number in parentheses corresponds to the number of cells per cluster. The dot size represents the percentage of cells expressing the marker, and the color intensity (from light to dark red) indicates the averaged normalized expression (log normalized counts) of a given marker within a given cell cluster. Additional differentially expressed genes between clusters are shown in Data S1.

Figure S4

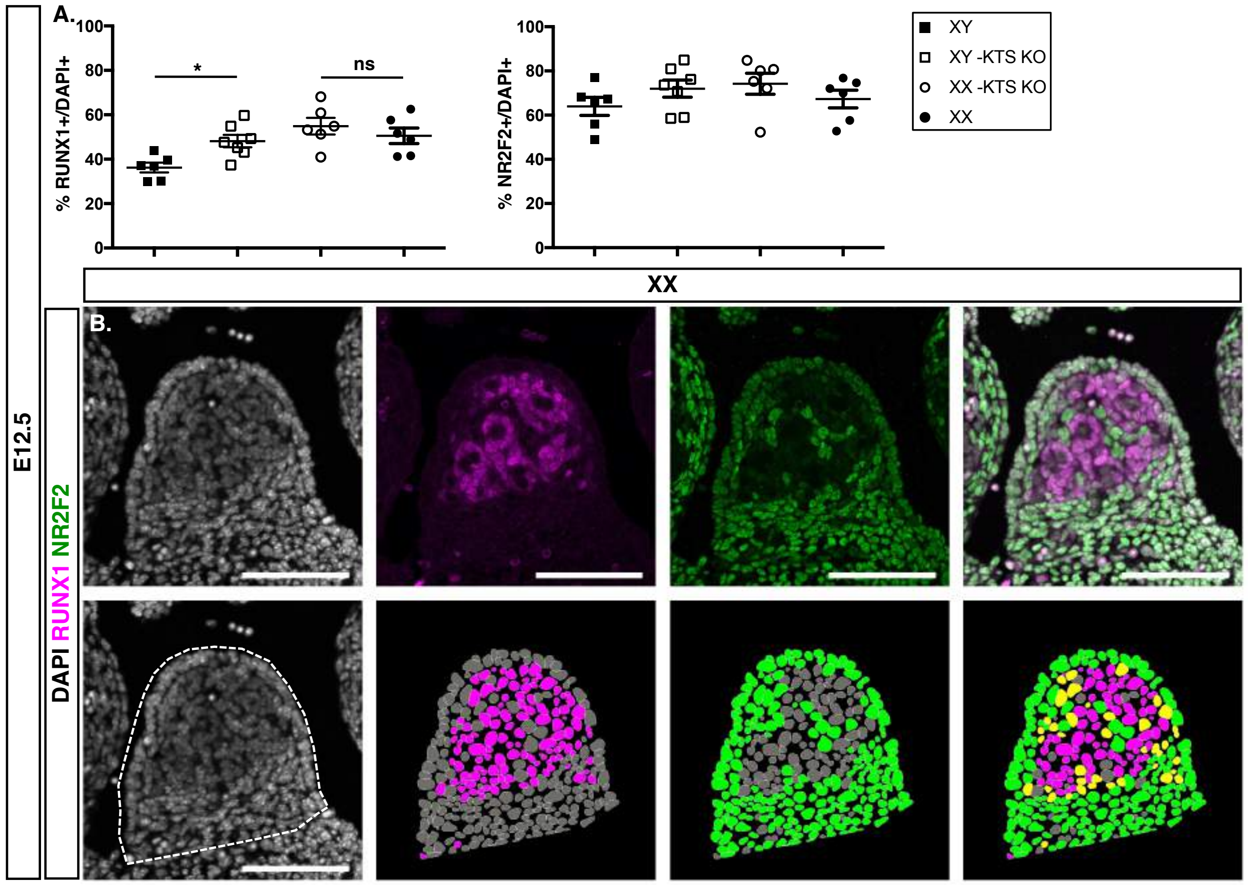

Fig. S4. -KTS is necessary for sex differentiation of the supporting cells.
(A) Quantification of RUNX1+ and NR2F2+ cells normalized to DAPI+ cells in XY and XX wildtype and XY and XX *-KTS KO* gonads. n=3-4 biological replicates, 2 gonads/embryo. Data are shown as means ± SEM. **(B)** Immunodetection of the pre-supporting and pre-granulosa cell marker RUNX1 (magenta) and the progenitor marker NR2F2 (green) positive cells and image processing steps used for RUNX1 and NR2F2 positive cells quantification shown in a XX gonad as described in materials and methods. Scale bars: 100 µm. Nuclei labelled with DAPI are shown in gray. Upper panel: original images; lower panel: corresponding processed images: manual drawing of gonad outline on DAPI channel to determine the Region Of Interest of the gonad (left panel), threshold adjustment of RUNX1 positive (magenta) and negative (gray) cells (second panel), threshold adjustment of NR2F2 positive cells (green) and negative (gray) cells (third panel), and combination of RUNX1 (magenta), NR2F2 (green), double positive (yellow) cells, and double negative (gray) cells (last panel).

Figure S5

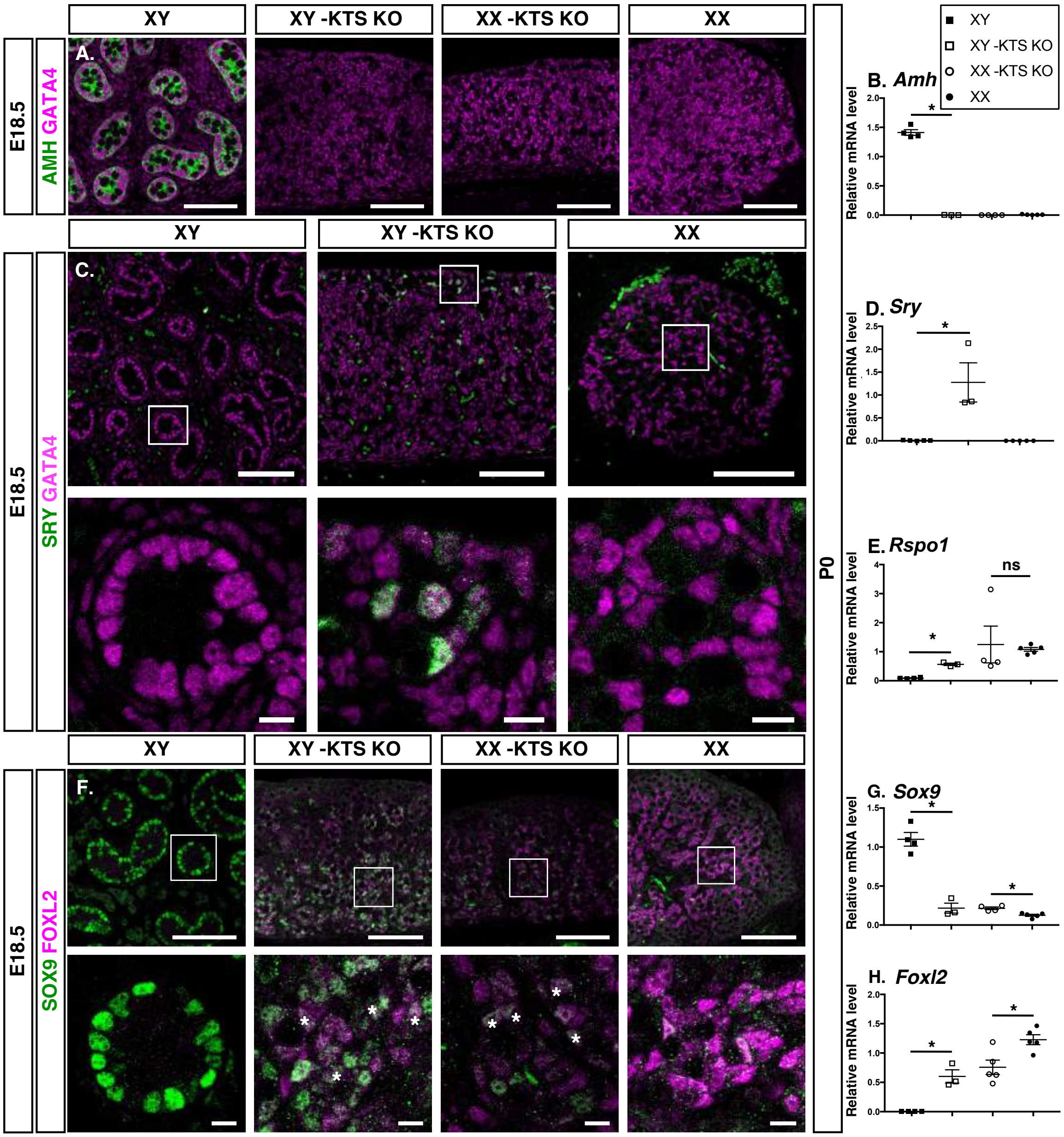

Fig. S5. Gonadal dysgenesis in *-KTS KO* gonads around birth.
(A) Immunofluorescence of the Sertoli cell marker AMH (green) and GATA4 (magenta) in indicated genotypes at E18.5. Scale bars: 100 µm. **(B)** RT-qPCRs show relative mRNA expression of *Amh* normalized to *Gata4* in indicated genotypes at P0. **(C)** Co-immunostaining of SRY (green) and the gonadal marker GATA4 (magenta) at E18.5 (scale bars: 100 µm, 10 µm). RT-qPCRs show relative mRNA expression of *Sry* **(D)** and *Rspo1* **(E)** normalized to *Gata4*. **(F)** Immunodetection of the Sertoli cell marker SOX9 (green) and of the pre-granulosa cell marker FOXL2 (magenta) (scale bars: 100 µm, 10 µm) reveal double positive cells (white asterisks) in -*KTS K*O gonads. The acquisition settings for the fluorescent signals are the same for all panels. **(G-H)** Quantification of *Sox9* and *Foxl2* transcripts after normalization to *Gata4* by RT-qPCR. **(A, C, F)** Data are representative of triplicate biological replicates. **(B, D-E, G-H)** Data are shown as means ± SEM.

Figure S6

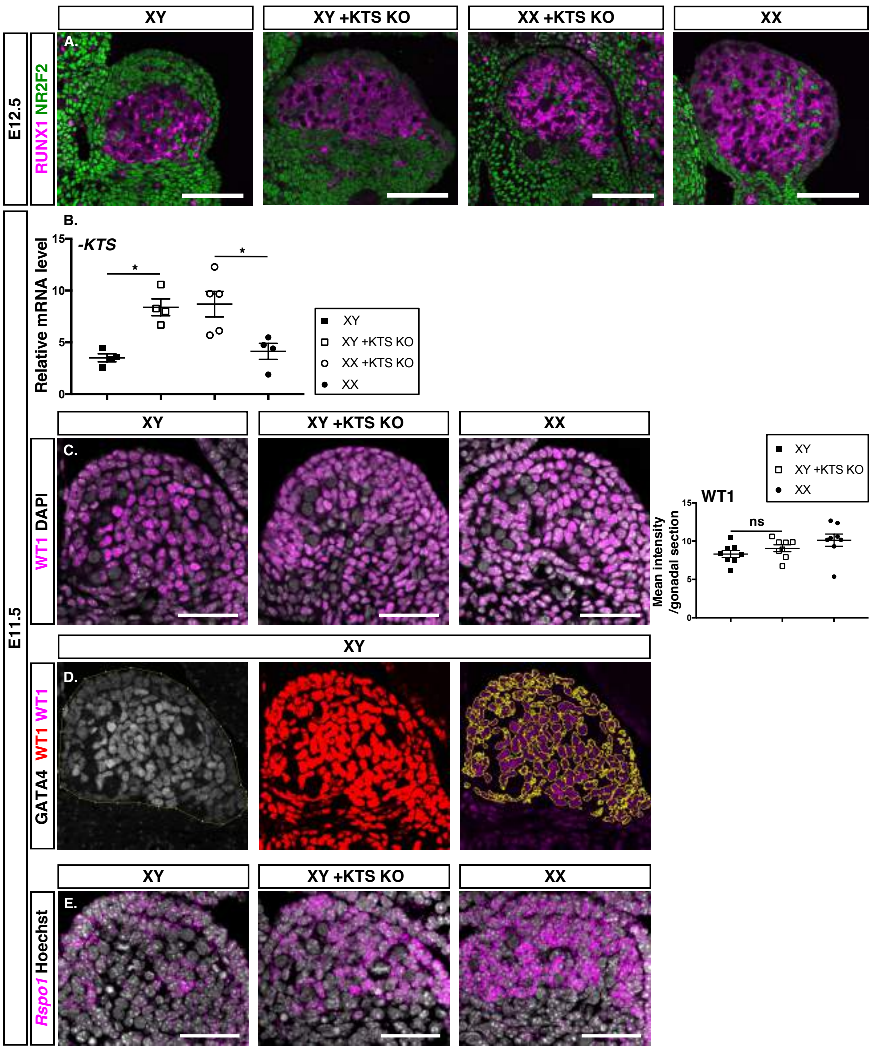

Fig. S6. Increased amounts of -*KTS*/-KTS in absence of +KTS.

(A) Immunodetection of the pre-supporting and pre-granulosa cell marker RUNX1 (magenta) and the progenitor marker NR2F2 (green) at E12.5 showing abundant NR2F2-positive cells under the coelomic epithelium in XY gonads, a hallmark of developing testis. This enrichment in NR2F2-positive cells was not detected in XX and XY and XX *+KTS KO* gonads (n=4 biological replicates). Scale bars: 100 µm. **(B)** Quantification of *-KTS* transcripts in XY, XX wildtype, and *+KTS KO* gonads by RT-qPCR (normalized to *Gapdh*) at E11.5 (20-21ts). Data are shown as means ± SEM. **(C)** Immunodetection and quantification of the signal intensity of WT1 (magenta) in XY and XX wildtype and in XY *+KTS KO* with two alleles only encoding *-KTS* (n=4 biological replicates). Scale bars: 50 µm. Nuclei labelled with DAPI are shown in white. Data are shown as means ± SEM. **(D)** Image processing steps for WT1 signal intensity quantification shown with the XY gonad. Left panel: Manual drawing of gonad outline on GATA4 channel to determine the Region Of Interest of the gonad (ROI-gonad). Middle channel: Automatic threshold adjustment of WT1 signal allowing determination of ROI-WT1. Right panel: Visualization of ROI-WT1 in ROI-gonad (yellow) giving the gonadal WT1 signal (magenta) as described in materials and methods. **(E)** RNAscope in situ hybridizations revealed *Rspo1* expression in XY *+KTS KO* gonads at E11.5 (n=3 biological replicates). Scale bars: 50 µm. Nuclei labelled with Hoechst are shown in white.

Figure S7

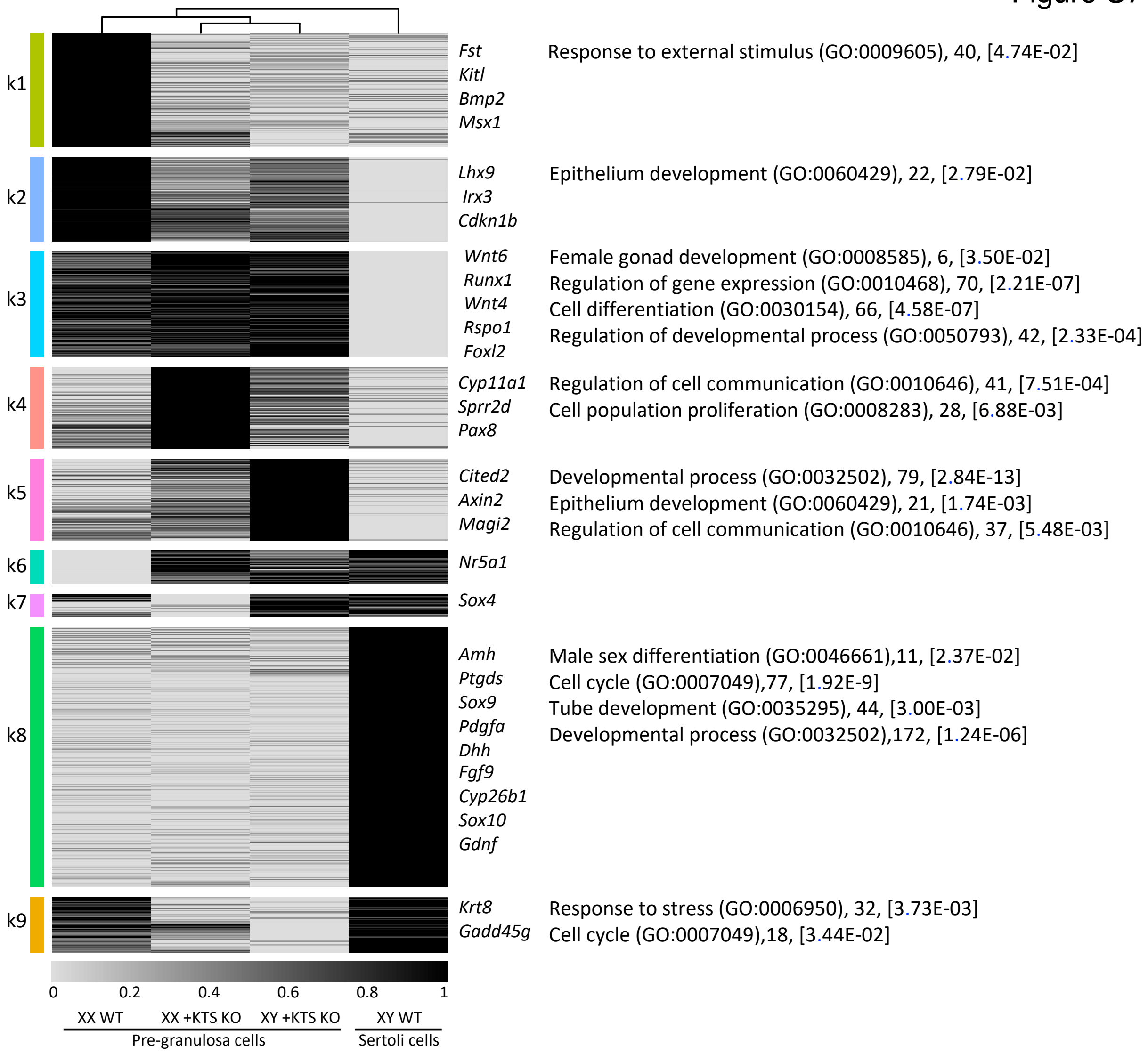

Fig. S7. Comparison of wildtype pre-granulosa and Sertoli cells with XY and XX +*KTS KO* pre-granulosa cells.

Heatmap showing the expression of the top 1,189 differentially expressed genes from Sertoli cells of XY wildtype (cluster 12, see Fig.1D-E), XY and XX +*KTS KO* pre-granulosa cells (c10, c33) and wildtype pre-granulosa cells (c5, c25). These genes were classified by expression patterns (k1-k9) (Data S2). Known marker genes and significantly enriched biological processes (Data S3) are shown for each expression pattern on the right-hand side. The dendrogram between the indicated genotypes shows the similarity of +*KTS KO* pre-granulosa cells and wildtype pre-granulosa cells. The grayscale intensity (from white to black) indicates the averaged normalized expression of a given gene within a cluster.

Figure S8

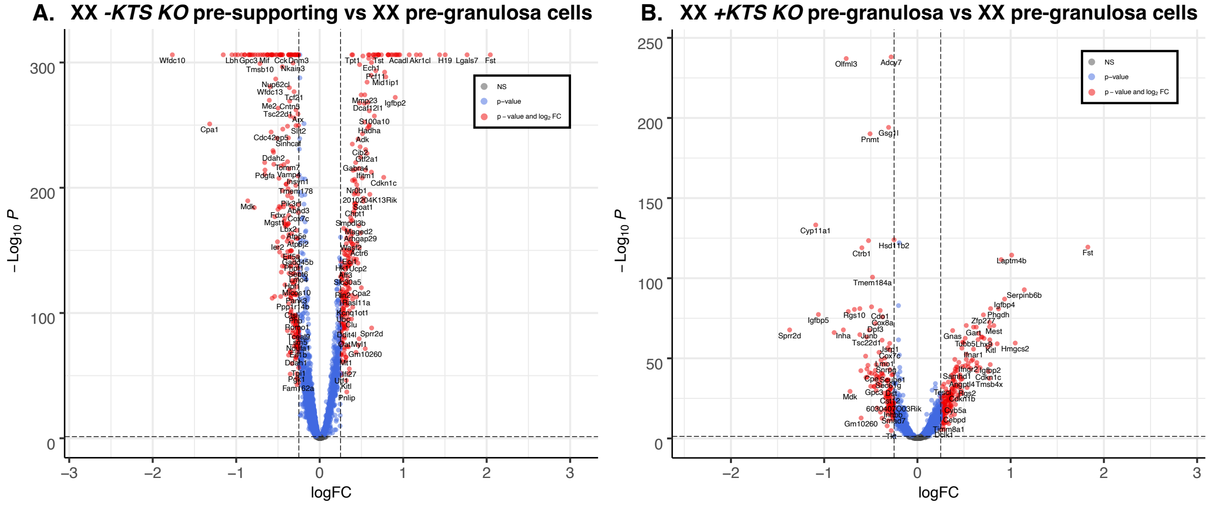

Fig. S8. Differential gene expression analysis between XX *-KTS KO* pre-supporting cells or XX +*KTS KO* pre-granulosa cells and XX pre-granulosa cells at E12.0.
(A) Volcano plots of differential gene expression in XX *-KTS KO* pre-supporting cells (including 1616 XX *–KTS KO* cells from cluster c3, 4 from c15 and 1 from c31) or **(B)** XX +*KTS KO* pre-granulosa cells (including 233 XX +*KTS KO* cells from c33, 59 from c10, 5 from c5 and 2 from c25) and XX pre-granulosa cells in XX control gonads (including 3535 XX control cells from c5, 860 from c25, 10 from c10 and 1 from c33). Red dots correspond to genes deregulated more than 1.2-fold. In XX *-KTS KO* pre-supporting cells, the expression of 319 genes was significantly deregulated (FDR adjusted p-value ≤ 0.05) (Data S4).

Figure S9

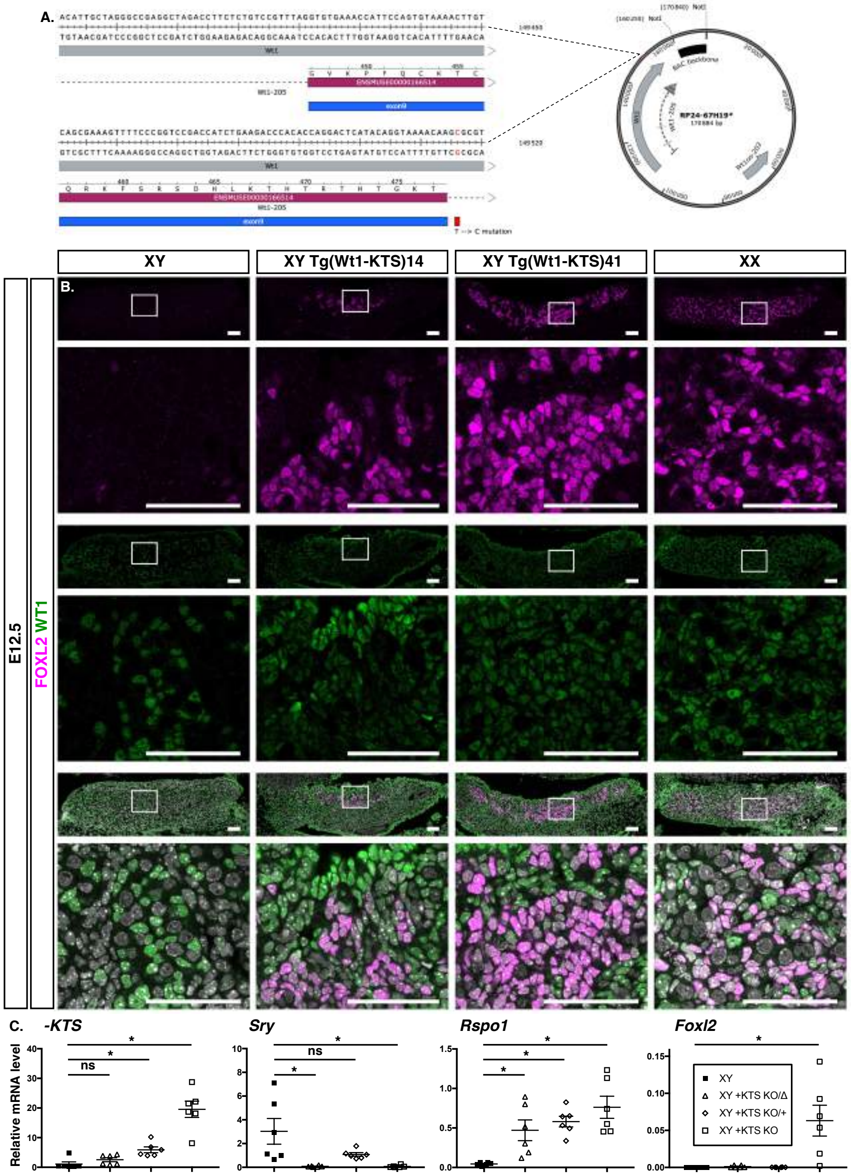

Fig. S9. -KTS promotes ovarian differentiation.

(A) Introduction of a point mutation in the BAC *Wt1*, RP24-67H19 to ablate synthesis of +KTS variants. **(B)** Immunodetection of WT1 (green) and FOXL2 (magenta) for each channel separately in gonads of two transgenic embryos *Tg(Wt1-KTS) 14* and *41*. On the merged images, nuclei labelled with Hoechst are shown in white. Scale bars: 50 µm. **(C)** Quantification of *-KTS, Sry, Rspo1,* and *Foxl2* transcripts in XY wildtype (both alleles produced +*KTS* or -*KTS*), XY +*KTS KO/Δ* (one allele produced -*KTS*), XY +*KTS KO/+* (one allele produced -*KTS*, one allele produced +*KTS* or -*KTS*), and +*KTS KO* (both alleles produced -*KTS*) gonads by RT-qPCR (normalized to *Gapdh*) at E11.5 (20-21ts). -*KTS* levels correlate with the genetic ability to produce -*KTS*. *Sry* expression is low, and *Rspo1* remains expressed in all mutants, suggesting a delay in testis development. The up-regulation of *Foxl2* is only observed in +*KTS KO* gonads suggesting that -*KTS* must reach a threshold level to promote ovarian differentiation at E11.5. Data are shown as means ± SEM.

Table S1

Samples	Genotype	Tail somite	Number of cells
MTCR2	XY +/+	24	8,288
MTCR4	XY +/+	24	6,029
MTCR1	XX +/+	24/25	6,470
MTCR5	XX +/+	25	6,542
MTCR3	XY -KTS KO	24	5,575
	XY -KTS KO	24	
MTCR6	XY -KTS KO	20/21	5,247
	XY -KTS KO	23	
MTCR11	XX -KTS KO	23	4,479
MTCR12	XX -KTS KO	21	5,288
	XX -KTS KO	20	
	XX -KTS KO	25	
MTCR7	XY +KTS KO	26	6,910
MTCR9	XY +KTS KO	22	7,391
MTCR8	XX +KTS KO	23	8,700
MTCR10	XX +KTS KO	20	4,441

Table S1. Details of the samples collected for the single-cell RNA sequencing.
The first column lists the identification of the samples. For each embryo, both gonads dissected from the mesonephros were collected. In *-KTS KO* samples, gonads of two to three embryos were pooled together. The second column lists the genotype of the samples, the third column lists the number of tail somites (ts), and the last column lists the number of cells after removal of low quality ones and doublets.

Primer name	Application	Sequence (5’→3’)
Sry forward	Sex genotyping	GTC ACA ATT GTC TAG AGA GC
Sry reverse	Sex genotyping	ACT GCA GAA GGT TGT ACA GT
CCR5mL	Genotyping internal control	CAA CCG AGA CCT TCC TGT TC
CCR5mR	Genotyping internal control	ATG TGG ATG GAG AGG AGT C
Wt1 Exon9F	Genotyping as described in (*20*)	GTG AAA CCA TTC CAG TGT AAA AC
Wt1 Intron9R		GCT CAT TGA CCC TTT CTC TG
Wt1 pgk	Wt1 KO genotyping	CTA CCG GTG GAT GTG GAA TGT GT
Wt1 shared	Wt1 KO genotyping	TCC CGA ACA ATT TCA CCT TGA ATC
Wt1 Wt	Wt1 KO genotyping	AGC CTA ACT TTG GGG CTT ATC TCC
Foxl2	RT-qPCR	GGC GTC GTG AAC TCC TAC A
	RT-qPCR	TGC AGA TGA TGT GCG TGA G
Sox9	RT-qPCR	GTA CCC GCA TCT GCA CAA C
	RT-qPCR	CTC CTC CAC GAA GGG TCT CT
Amh	RT-qPCR	CCC TTC AAC CAA GCA GAG AA
	RT-qPCR	AGT CAT CCG CGT GAA ACA G
Rspo1	RT-qPCR	CGA CAT GAA CAA ATG CAT CA
	RT-qPCR	CTC CTG ACA CTT GGT GCA GA
Sry	RT-qPCR	AGC CTC ATC GGA GGG CTA
	RT-qPCR	AGG CAA CTG CAG GCT GTA AA
Wt1	RT-qPCR	CAT CTG ATT CCA GGT CAT GC
	RT-qPCR	GAG GAC GCC CTA CAG CAG
+KTS	RT-qPCR	CAC CAA AGG AGA CAC ACA GGT
	RT-qPCR	TTC ACT TGT TTT ACC TGT AT
+KTS mutated (*)	RT-qPCR	CAC CAA AGG AGA CAC ACA GGT
	RT-qPCR	TTC ACT TGT TTT C*CC G*GT AT
-KTS	RT-qPCR	CAC CAA AGG AGA CAC ACA GGT
	RT-qPCR	GGG CTT TTC ACC TGT ATG AG
Gata4	RT-qPCR	GGA AGA CAC CCC AAT CTC G
	RT-qPCR	CAT GGC CCC ACA ATT GAC
Gapdh	RT-qPCR	GTG TTC CTA CCC CCA ATG TG
	RT-qPCR	GTC ATT GAG AGC AAT GCC AG

Table S2. List of primers

Antibody name	Reference	Dilution	Source
RUNX1	ab92336	1/500	Abcam
NR2F2	PP-H7147-00	1/200	R&D Systems
FOXL2	NB100-1277	1/100	Novus Biologicals
FOXL2		1/200	kindly provided by Dr. Dagmar Wilhelm
SOX9	HPA001758	1/250	Sigma-Aldrich
AMH	Sc6886	1/100	Santa Cruz Biotechnology
SRY		1/200	kindly provided by Dr. Dagmar Wilhelm
GATA4	Sc1237	1/200	Santa Cruz Biotechnology
WT1	M3561	1/300	Agilent Dako
WT1	AF5729	1/300	R&D Systems

Table S3. List of antibodies.

Data S1. Differentially expressed genes between clusters of +*KTS KO*, -*KTS KO* and wild-type XY and XX gonads.

Data S2. Differentially expressed genes between clusters k1 to k9 related to the heatmap in Fig. S7.

Data S3. Biological processes through a Gene Ontology and pathway enrichment test related to the heatmap in Fig. S7.

Data S4. Differential gene expression analysis between XX -*KTS KO* pre-supporting cells or XX +*KTS KO* pre-granulosa cells and XX pre-granulosa cells at E12.0.
